# Decentralized restoration of distribution systems with coupling neighboring microgrids

**DOI:** 10.1016/j.heliyon.2024.e28344

**Published:** 2024-03-22

**Authors:** Qi Cui, Feng Liu

**Affiliations:** The College of Economics and Management, Shenyang Agricultural University, Shenyang 111000, LiaoNing, China

**Keywords:** Coupling neighboring microgrids, Decentralized technique, Distribution network restoration, Multiagent system

## Abstract

In this study, a multi-agent system (MAS) is incorporated in a decentralized strategy to restore distribution systems while taking into account coupling neighboring microgrids (CNMGs). This provides modeling for renewable energy sources (RESs), electric vehicles (EVs), battery storage systems (BSS) and load. The desired and most favorable restoration path is found by the MAS, in which zone agents are dispersed across the distribution system. The MAS can also manage microgrids (MGs) overloaded as the unbalance operation of RESs, BSS, EVs, and load. This is realized by making a bridge between MGs and neighboring non-overloaded MGs. The suggested method adheres to voltage and power flow restrictions while operating according to expert system standards. The recommended approach is put to the test using a 33-bus radial distribution system. MATLAB calculations on agents and power flow are carried out in order to verify the validity of the choices made by agents. The proposed restoration plan is able to obtain the best power supply path with a low number of switching in the event of a fault so that the voltage magnitude is higher than 0.9 p.u. and free capacity is available for the distribution lines. The smart charging strategy of EVs reduces 93% of their turn off compared to the non-smart charging strategy. However, if the CNMG plan is established, all vehicles can be powered.

## 1 Introduction

### 1.1 Motivation and research literature

Islanded MGs may be met by renewable energy sources (RESs) such photovoltaic (PV) systems and wind energy. Nonetheless, the MG is vulnerable to overflow because to the sporadic nature of RESs and load. To address the problem, a number of studies have suggested using energy storage systems (ESS), grid-linked MGs, adjacent MGs (CNMGs), and non-renewable energy sources (NRESs) such diesel generators and microturbines [1,2]. It should be noted that using ESSs or NRESs in a lone MG may result in higher energy prices because to their operating expenses [3]. Furthermore, compared to an isolated MG, as the demand within the distribution system escalates, the flexibility of the system decreases owing to an increase in unknown parameters, rise in distribution network operating cost, and so forth [4]. However, based on [5], connecting nearby MGs can be assumed as a reasonable and lucrative strategy for MG overload management. This approach was established in Ref. [5], which states that individual MGs can be fed via their nearby MGs during overload conditions. Because every two nearby MGs have a normally-open interconnecting static switch (ISS), the coupling neighboring MGs approach may be done by shutting the ISS in an overload state.

A new structure of CNMGs is introduced in Ref. [6] so that a distribution system is healed by itself when faults occur. Moreover, Ref. [7] adopts the concept of CNMGs to describe an overload management method, where the authors use a specific decision-making algorithm that is dynamic and is based on several criteria. Additionally, the dynamic operation of DGs in a CNMG configuration is discussed [8] and the dynamic security of CNMGs is evaluated [9]. The coupling of far-located droop-regulated MGs at deficient power periods becomes straightforward and facilitated using a supervisory control design [10].

According to a survey by the US Department of Energy (DOE), distribution systems are responsible for the majority of power system disruptions [11]. As a result, distribution system restoration might aim at recovering the largest potential disrupted loads of a defective feeder [12]. Restoration can be implemented via either centralized or decentralized coordinating systems. The former contains optimally determines the best restoration path [13], but because this strategy relies on a central unit to make decisions and the process is slow and insecure [14]. The decentralized technique, on the other hand, is dependent on heuristic algorithms or expert system principles, and therefore necessitates careful design, as seen by the speedy restoration [15].

Various restoration approaches in the distribution network are being researched [16]. describes the multi-agent system (MAS), which is a decentralized technique that employs expert system principles to discover a restoration path. Furthermore, this strategy employs a network of agents that interact with one another to arrive at a good choice [16]. The best repair path is determined by the authors of [17] using the Artificial Bee Colony (ABC) approach, which is based on a MAS with five different kinds of agents. Explains a decentralized approach based on MAS for DG and EV-based distribution network maintenance in Ref. [18]. Ref. [19] also presents a hierarchical coordination mechanism based on presumed DGs and load priority for distribution system restoration in distribution grids. In order to identify the optimal repair path [20], places four zone agents along each feeder and offers a MAS based on expert system principles. A decentralized MAS for distribution network repair in an ambiguous environment is provided by Ref. [21]. In Ref. [22], a distributed secondary consensus fault-tolerant control (FTC) technique for multi-agent microgrids (MG) is proposed. In the presence of MG faults, the suggested controller is used to correct for inaccuracies in the system frequency and voltage profiles. The suggested controller also allows the system to provide precise power sharing across the linked distributed generators (DGs) in MG. A coordinated smart building energy-sharing strategy for smart neighborhood buildings that are linked to internal energy storage devices and renewable energy sources is described in Ref. [23]. By increasing the use of locally produced renewable energy, this neighborhood energy management strategy seeks to reduce total power costs for all residents of smart buildings within the area. In the first step, an Improved Butterfly Optimization Algorithm (IBOA) determines a set of ideal consumption plans for every HEMS. In the second phase, a neighborhood energy management system (NEMS) is built using a consensus mechanism. Lastly, Table 1 displays the taxonomy of current research initiatives.

**Table 1:** Taxonomy of recent research works.

Ref.	Modeling of CNMG	Restoration method	Network limits
[5]	CNMG include load and RES	✗	✓
[6]		✗	✓
[7]		✗	✓
[8]		✗	✓
[9]		✗	✓
[10]		✗	✓
[13]	✗	Centralized method including Optimization method	✗
[14]	✗		✗
[16]	✗	Decentralized method based on MAS	✗
[17]	✗		✗
[18]	✗		✗
[19]	✗		✗
[20]	✗		✓
[21]	✗		✓
[22]	✗		✓
[23]	✗		✓
**Current paper**	**CNMG includes load, renewable generation, Fixed and mobile storage**	**Decentralized method including MAS-based ADRT**	✓

### 1.2 Research gaps

According to the above-mentioned studies, there are three major study gaps in the literature regarding restoration of network and CNMGs:

• Numerous studies [[13], [14], [15], [16], [17], [18], [19], [20], [21]] ignored CNMGs in favor of restoration techniques for distribution networks. The restoration service is required in the system when a failure occurs to increase the dependability of the distribution network and MGs, since it should be mentioned that CNMGs will be integrated in the distribution network's future design [9]. • Further research is included in the MG [[5], [6], [7], [8], [9], [10]], although it is anticipated that EVs will be covered in future MGs. • The MG model with RES, EVs, and load is required by the CNMGs program. The distribution system's limitations, including voltage and line flow limits, have not received much attention [16,17]. However, in power or distribution networks, these limitations are required.

### 1.3 Contributions

**Fig. 1:**
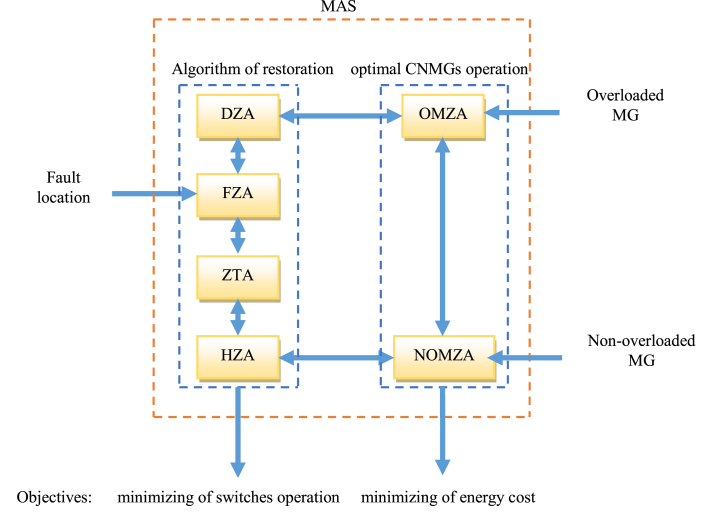
Proposed scheme of coupling of the CNMGs management and restoration algorithm.

To address the aforementioned concerns, this article employs a decentralized MAS-based approach for autonomous restoration of distribution systems with CNMGs, such as the RES, EV, BSS, and load models shown in Fig. 1. There is more than one zone agent between any two given switches in main feeds in the suggested restoration method: The faulty zone agent (FZA) is used as a decision-making agent by the faulted zone agent (FZA). The down zone agent (DZA) is used in zones that experienced power outages due to the problem. The agent of the healthy zone that has a tie switch attached to it is known as the zone tie agent (ZTA). For the zones in the healthy feeder along the restoration pathway, use this agent, also known as the healthy zone agent (HZA). Restrictions on the distribution network are considered in HZA and FZA. The CNMGs also use the overloading management approach, which employs MG switches and the MAS technique to link overloaded and non-overloading MGs. MG zone agents (MZA), OMZA (overloading MZA), and MZA (non-overloading MZA) are two different kinds of zone agents for each MG (NOMZA). To find a suitable restoration route in the distribution network and manage overloaded MGs with non-overloading MGs in the presence of CNMGs, the OMZA (NOMZA) coordinates with the DZA (HZA), as illustrated in Fig. 1. To lower switch operation and energy costs, the MAS includes an overloading control and restoration method, per Fig. 1. Finally, the following are the important contributions of this paper:

- In the presence of CNMGs, implement a decentralized method based on MAS for distribution network repair,
- Considering future MGs' models, which incorporate RES, EV, BSS, and load,
- In the repair process, take into account the limitations of the radial distribution system, and
- Load shedding rate is reduced as a result of the management of overloaded MGs with non-overloading MGs.

### 1.4 Paper organization

This is how the remainder of the paper is structured. In Section 2, the proposed decentralized MAS strategy for restoring a distribution system using CNMGs is explained. Numerical simulations are presented in Section 3, while the study's novelty are highlighted in Section 4 together with important findings from the conducted investigation.2. Problem model.

### 1.5 MAS architecture for distribution network restoration

**Fig. 2:**
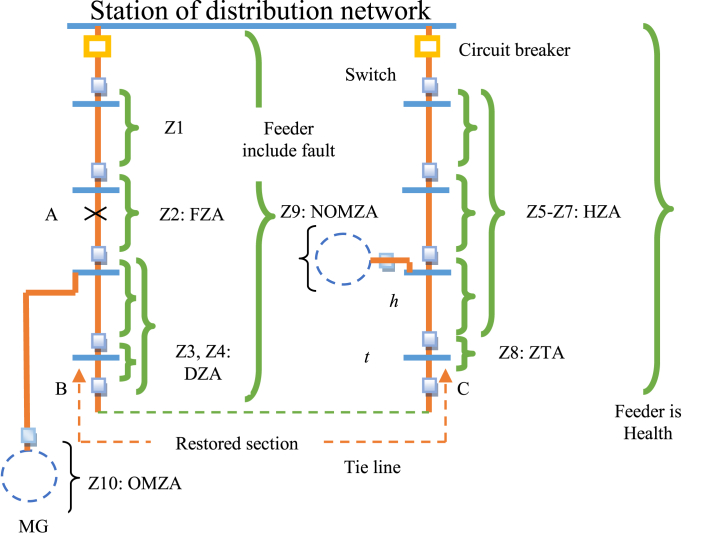
MAS Method for the proposed Restoration algorithm.

An internal zone fault and a tripped feeder circuit breaker signaled the start of the restoration process. In the first step, zones establish their status based on where the fault is located. In the second stage, they carry out their responsibilities using the MAS approach, which takes into consideration the necessary restoration aims and realistic constraints. For the distribution network, the MAS technique employed in this study incorporates four zone agents: FZA, DZA, ZTA, and HZA. The defective zone's decision-making agent is referred to as the FZA, the healthy zone's decision-making agent as the HZA, the healthy zone without a tie switch as the ZTA, and the zones that have lost power as the DZA. Furthermore, each MG on the network that is housed in a separate bus has two distinct kinds of zone agents, or MZAs: an overloaded MZA (OMZA) and a non-overloading MZA (NOMZA). Zone Z2 is a fault zone agent (FZA) because point A has a fault, and zone Z10 is an overload zone agent (OMZA) since the MG in this zone is overloaded, according to Fig. 2. The down zone agent categorization designates Z3 and Z4 zones as DZAs as well. Z8 is a ZTA because of its robust feeder and tie line connection. Lastly, Z5 through Z7 are HZA zone agents as they are situated on a healthy feeder. Because NOMZA does not experience overloading, it is also a Z9 zone. Communication between FZA and DZA, FZA and ZTA, ZTA and HZA, NOMZA and OMZA, MZA and DZA or HZA, and ZTA and HZA is also possible, as Fig. 2 shows. Throughout the restoration process, FZA collects and sends data to ZTA on DZA demand, including zone load, receiving power from NOMZA, receiving demand from OMZA, and obtaining generation power from FZA. ZTA is linked to DZAs with closed switches at points B and C in compliance with HZA standards or limitations, such as voltage and line flow limits, and FZA is isolated from the problematic feeder via Z2 via Z2. It is important to emphasize that OMZA receives demand, NOMZA receives producing power, and OMZA determines demand, with HZA choosing its status depending on zone load. The following technological constraints of the MAS method are also considered:

- Radial stricture of distribution network as Eq. (1):

(1) $$N_{line}=N_{bus}-1$$

where Nline and Nbus represent the respective amounts of feeder lines and feeder buses. Eq. (2) states the maximum current that may be used on all lines that connect the feeder.

(2) $$\left|I_{j}\right|\leq I_{\max\;j}$$

here *I*_*j*_ and *I*_max *j*_ are feeder line current and the maximum capacity of the feeder line.

- Bus voltage limit: The voltage limit must be satisfied for both defective and healthy feeder buses; hence, Eqs. (3), (4) yield the current limit corresponding to the voltage limit in the healthy feeder (Ivh) and faulty feeder (Ivf) [24,25].

(3) $$I_{vh}=\frac{V_{h}-V_{\min}}{Z_{h}}$$

(4) $$I_{vf}=\frac{V_{t}-V_{\min}}{Z_{f}}$$

where Ivh and Ivf are the maximum currents on healthy and faulty feeders, respectively, that may exist without exceeding the voltage limit. In addition, as can be seen in Fig. 1, Vt and Vh indicate the voltage magnitude of a healthy feeder in the tie bus and bus h, respectively, and their minimal voltage magnitudes. Vmin is the lowest allowed voltage magnitude, which is 0.9 per unit (p.u.) [[26], [27], [28]]. As seen in Fig. 1 [24], the impingence level Zh represents the amount of impedance between the substation and bus h. Furthermore, Zf's magnitude equals |Zst +0.5 (Zt|), where Zt is the restored section impedance [25] and Zst is the impedance in Fig. 2 between the substation and bus t. Additional information about the activities of zone agents is as follows: A) *Fault zone agent (FZA)*: If the following circumstances exist in the zone as a result of an inside fault, the FZA operation begins:

- The incoming current upstream (downstream) is substantially higher (near to) typical.
- The incoming current upstream and downstream is substantially higher than typical.

As a result, FZA initiates the restoration process and employs switches that are situated in this zone to isolate issues when the feeder circuit breaker triggers. In order to restore its up-stream zones in the next phase, the FZA additionally signals the feeder circuit breaker with information. It then establishes links with DZAs and ZTAs to determine the power consumption of these zones and the supply power that is available via tie lines. As a result, this zone agent is a decision-making agent that performs the following tasks: Step 1: Through a Request for Information (RFI) message, the FZA asks for the impedance and power needed by down zones, and the DZAs respond by giving the information to the FZA. Step 2: The ZTAs send Vbt, Zpt, and restoration permissible power after receiving CFP signals from the FZA and verifying the voltage limit of the healthy feeder (APh) in accordance with the ZTA operation. Step 3: Determine the highest amount of power that can be authorized from the healthy tie (APf) using Eq. (5), and determine the maximum power that can be allowed from the healthy tie (APp) by monitoring the voltage limitations in both the healthy and restored areas and utilizing FZA (APTi) as described in Eq. (6).

(5) $$AP_{f}=\left|V\right|\times \left|I_{vf}\right|$$

(6) $$AP_{Ti}=\min\left(AP_{hi},AP_{fi}\right)$$

where the voltage magnitude, denoted by |V|, is assumed to be one p.u. When compared to APT and the total power used by down zones as Eq. (7), FZA achieves group restoration with only one switching operation.

(7) $$\max_{i\in n_{T}}\left(AP_{Ti}\right)\geq \sum_{j=1}^{n_{z}}\left|S_{j}\right|$$

where the numbers for the tie and down zones are, respectively, nT and nz. When condition (8) is met, FZA sends a message of acceptance to ZTA with a maximum APT value in relation to other ZTAs, telling ZTA to shut its switch. Step 4: The zone restoration operation is carried out along the restoration route if group restoration is not possible, that is, if the total power demand by down zones above the maximum value of APT. Since a few down zones are being fed by healthy connections in this scenario, Eq. (8) is met.

(8) $$AP_{Ti}\geq \sum_{j=1}^{n}\left|S_{j}\right|$$

The amount of down zones that a healthy tie I can feed is indicated by the number n. In the end, the FZA tells a lot of down zones to open their switches and a lot of ZTAs to shut their switches by sending an accept proposal message. B) Down zone agent (FZA): The DZA operation notifies the FZA of its power need and impedance after first receiving the RFI message from the FZA. Moreover, it receives a periodic command from FZA to turn on its zone restoration switch. Furthermore, in order to get zone demand or generation power from MG, DZA uses RFI signals to notify MZA if CNMG needs overloading control. C) ZTAs, or zone tie agents: ZTA functions as follows: **Step 1:** Receiving of CFP massage from FZA.

Step 2: ZTA sends the RFI massage to HZAs placed along the healthy feeder's path for restoration. The HZA then sends each healthy zone's Vh, Vmin, Zh, and spare capacity, which are computed as Eq. (9):

(9) $$I_{ava\;j}=\min_{k}\;\left(I_{\max\;k-j}-I_{\;k-j}\right)$$

where Iava j, Imax k-j, and Ik-j stand for, in that order, the branch k's current magnitude, the maximum current carrying capacity of zone j, and the available spare current. Step 3: To determine Ivh and the lowest value of Iava, denoted by Ich, ZTA derives Eq. (10) and applies Eq. (3).

(10) $$I_{ch}=\min_{j}\;\left(I_{ava\;j}\right)$$

Therefore, the *AP*_*h*_ for individual ZTAs is calculated in Eq. (11):

(11) $$AP_{h}=\left|V\right|\times \left|I_{spare}\right|\;\forall I_{spare}=\min\left(I_{vh},I_{ch}\right)$$

With |V| equal to one p.u., Ispare shows the spare current that can be restored within the safe voltage range of the healthy feeder. After sending the values of Vt, Zst, and APh to FZA, if ZTA is chosen for group restoration, it shuts off its switch and waits for FZA to send the accept proposal message. D) Healthy Zone Agent, or HZA: After receiving the RFI message from the ZTA initially, the HZA process uses equation to determine Iava j. (9). Finally, but just as importantly, it gives ZTA data on Vh, Vmin, Zh, and Iava j. Additionally, if CNMG requires overloading management, HZA transmits RFI signals to MZA in order to collect zone demand or MG supplying power.

### 1.6 Overloading management of CNMGs

**Fig. 3:**
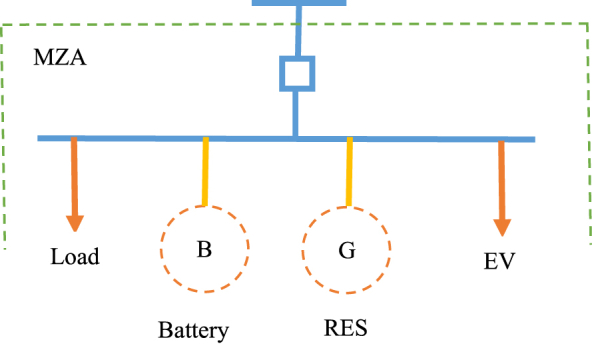
The proposed framework of MG. A) *The MG elements model*: In the proposed MG, the RES system is a power source, and the output power of wind and PV systems relies on wind speed and irradiation. To put it another way, the power of a wind unit (PW) is calculated as Eq. (12) [29], whereas the power of a photovoltaic system (PPV) is calculated as Eq. (13) [29].

Fig. 3 depicts the suggested MG structure, which incorporates renewable energy sources (RESs), electric vehicles (EVs), battery storage systems (BSS), and load. The RES also contains a solar system and a wind turbine. Furthermore, each MG has a zone agent known as the MG zone agent (MZA), which coordinates with the MZA of other MGs as well as the distribution system zone agents like FZA, DZA, ZTA, and HZA. Finally, the CNMGs overloading management approach supports distinct zone agents' interoperability.

(12) $$P_{W}=\left\{ \begin{array}{c} \begin{array}{cc} 0\; & v\leq v_{ci} \end{array}\\ \begin{array}{cc} P_{r}.\frac{v-v_{ci}}{v_{r}-v_{ci}} & v_{ci}\leq v\leq v_{r} \end{array}\\ \begin{array}{cc} P_{r}\; & v_{r}\leq v\leq v_{co} \end{array}\\ \begin{array}{cc} 0\; & v_{co}\leq v \end{array} \end{array}\right.$$

(13) $$P_{PV}=R\times A\times \eta$$

V, vci, vr, and vco, respectively, stand for wind speed, cut-in, rated, and cut-out wind speed. Pr is also short for a wind turbine's rated power. Solar radiation, photovoltaic area, and overall efficiency of photovoltaic panels and DC/DC converters are also represented as *R*, *A* and *η* [29]. Finally, the RES (PRES) output power is equal to P_W_ + P_PV_.

The total power of loads (L) and electric cars is added to determine the MG power demand (LEV). The two forms of charging procedures for electric cars are controlled and unmanaged charge schemes, it should be noted. EVs charged at peak load hours in a mismanaged charge plan, and EVs charged at low load times under a managed charge strategy [[30], [31], [32], [33], [34]]. Finally, LMG equals *L + L*_*EV*_ for the MG power demand.

In addition, MG is involved in the following two scenarios:

- If *P*_*RES*_ ≥ *L*_*MG*_, thus, the excess of generation, i.e. P_RES_ – L_MG_, is stored in the battery, or is sent to other CNMGs, or is stored in the battery and sent to other CNMGs. Therefore, the stored energy of battery (*E*) at time *t* calculates as Eq. (14).

(14) $$E_{t}=E_{t-1}+\eta_{ch}P_{ch}\;\forall 0\leq P_{ch}\leq P_{RES}-L_{MG}$$

where *P*_*ch*_ is the charging power of the battery.

- If *P*_*RES*_ ≤ *L*_*MG*_, thus, the deficiency of demand, i.e. *L*_*MG*_ - *P*_*RES*_, is provided by the battery or other CNMGs. Therefore, the stored energy of the battery calculates as Eq. (15).

(15) $$E_{t}=E_{t-1}-\frac{1}{\eta_{dch}}P_{dch}\;\forall 0\leq P_{dch}\leq L_{MG}-P_{RES}$$

where *η*_*ch*_ and *η*_*dch*_ are battery efficiency in charging and discharging mode, respectively. Also, *P*_*dch*_ is the discharging power of the battery. It should be noted that the battery's capacity is Emax, meaning that, in most cases, its stored energy exceeds Emin (the minimum stored energy) and is less than Emax. Hence, MG gets electricity from CNMGs if the battery's discharge power is insufficient to cover the whole amount of the demand shortfall. B) *Micro-grid zone agent (MZA) operation*: The two types of MG zone agents are overloading MZA (OMZA) and no overloading MZA (NOMZA). When overloading happens in certain MGs, such PRES + Pdch ≡ LMG, these MGs' MZA is OMZA whereas other MGs' MZA is NOMZA. Consequently, each OMZA uses Eq. (16) to ascertain the overloading value of each MG (Pover) in the first stage:

(16) $$P_{over}=L_{MG}-P_{RES}-P_{dch}$$

In the subsequent phase, OMZAs communicate with NOMZAs using RFI signals to allow NOMZA to ascertain the excess generation of MGs (Pexc) without being overburdened. In addition, the following formula, Eq. (17), is used to determine Pexc:

(17) $$P_{exc}=P_{RES}-P_{ch}-L_{MG}$$

Finally, NOMZA compared Pover and Pexc values, and if Pexc ≥ Pover for two CNMGs, NOMZA closed its switch and signaled OMZA to do the same. Additionally, MZA works with DZA and HZA when overload management and restoration are needed at the same time.

A mathematical model is included into the plan that is being considered [[35], [36], [37], [38], [39]]. The optimization formulation [[40], [41], [42]] serves as the foundation for the mathematical model in this work. An objective function is a component of an optimization model [[43], [44], [45]]. The objective function has a min (max) phrase that indicates the function's minimum (highest) value [46,47]. It might be described as a function that has one or more goals. The optimization issue includes the different limits [[48], [49], [50], [51], [52]]. Limitations are expressed as equality and inequality [[53], [54], [55], [56], [57]]. Models that are linear, non-linear, mixed integer linear, or mixed integer non-linear are the constraints [58]. Intelligent devices are required to apply the optimization model on a network or hybrid system [[59], [60], [61], [62], [63], [64], [65]]. The foundation of smart systems is telecommunications technology. These systems coordinate the different power components. System processing is quick under these conditions, and network administration is easy.

## 2 Numerical results

### 2.1 Case study

**Fig. 4:**
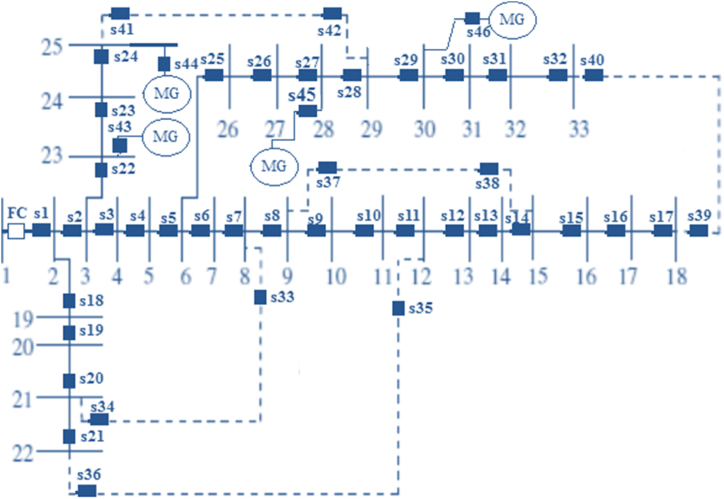
Distribution network of 33-bus [58].

Four MGs are anticipated to be part of this network; they may be found on busses 23, 25, 28, and 30. The load of each MG is equivalent to the load of the bus when a bus and an MG are in the same location. Additionally, even though the EV charging rate is 3 kW, the EV numbers for the MGs in buses 23, 25, 28, and 30 are 21, 60, 21, and 30, respectively, [58]. The 400, 900, 400, and 500 kWh batteries in these MGs are also said to have an efficiency of 0.88 [59]. These MGs can generate 330, 800, 330, and 510 kW of power from wind systems and 260, 720, 260, and 330 kW of power from solar systems, respectively (see Fig. 4).

### 2.2 Results

The suggested restoration issue is developed in MATLAB 2015b to investigate the problem's capabilities in several scenarios:

A) *Network restoration without taking into account CNMGs*: In the context of a 33-bus network, this section explores the possibilities of the suggested restoration technique in the absence of CNMGs. In the case of a failure, the circuit breaker for the 33-bus network will trip first, cutting off the FZA from the rest of the network by opening switches for the problem site. When FC receives a signal from FZA, FC reacts by reopening FZA's upstream zones. Thus, in order to determine the total DZA load in the first phase, FZA starts the restoration process by getting in touch with DZA using RFI signals. The results of the first stage in Table 2 show that when a problem occurs in the last zone of a feeder, as Zone 18 in the system provided, the restoration method is not necessary. Since there is no DZA in this situation, there is no load. Furthermore, if FZA is regarded as zone (bus) 19, 27, and 14 respectively, the total load of DZA is 295.5, 1204.2, and 284.6 kVA. FZA 19, 27, and 14 may now access tie lines (21, 8) and (22, 12), tie lines (25, 29), and tie lines (15, 9), (12, 22), and due to the effective restoration of the network (18, 33). ZTA transmits an RFI message to HZA in order to get Vbh, Vmin, Zph, and Iava, and FZA sends a CFP message to ZTA in order to calculate its allowable power based on the bus voltage and line current limits. Table 3 provides an overview of the outcomes from this section. For fault locations 19, 27, and 14, two tie lines are chosen using the computation approach shown in Table 3. (5). (12). Table 3 displays the open/closed switches for the various problem sites. For FZAs 19 and 14, two (four) switches opened and closed; whereas, for FZA 27, four (4) switches opened and closed. In situations where the issue site is between 19 and 14, the balance between generation and loads may also be used to satisfy the restoration approach. During the FZA 27 restoration phase, the planned network does not offer 632.5 kVA of DZA load in order to balance supply and demand. as the tie lines' maximum power is insufficient to meet the DZA standard. Following the restoration operation, the results of the load flow are shown in Table 4, along with the maximum voltage variation and the minimum permissible current (MAC) that may flow from distribution lines. These factors were chosen as follows:

**Table 2:** DZA demand based on different FZA locations.

Faulty bus	DZA (buses)	DZA demand (kVA)	Available tie line
18	–	0	–
19	20–22	295.5	(21, 8), (22, 12)
27	28–33	1204.2	(25, 29), (18, 33)
14	15–18	284.6	(15, 9), (12, 22), (18, 33)

**Table 3:** Optimal restoration situation based on different FZA locations.

FZA location	Optimal tie line/maximum power of tie line (kVA)	Switches in open mode	Switches in close mode	Total number of busses	Total load not supplied without considering FZA load (kVA)
18	–	s17	–	32	0
19	(21, 8), (22, 12)/213.3, 103.5	s18, s19	s33-s36	32	0
27	(25, 29), (18, 33)/425.2, 216.7	s26, s27, s29, s30	s39-s42	31	632.5
14	(15, 9), (12, 22),/180.3, 103.5	s13, s14	s35-s38	32	0

**Table 4:** Values of MVD and MAC for different FZA locations based on load flow results after the restoration process.

FZA location	MVD (p.u.)	MAC (p.u.)
18	0.085	0.021
19	0.081	0.036
27	0.076	0.011
14	0.082	0.028

Maximum voltage deviation (in per unit) = 1 – min (voltage of all busses)

MAC (in per unit) = min (maximum line current– line current flow)

Given that the lowest and highest voltages are 0.9 and 1.05 p.u., respectively, the allowed voltage fluctuation is 0.1 p.u. (1–0.9 = 0.1). The highest voltage variation for fault sites 18, 19, 27, and 14 is, respectively, 0.085, 0.081, 0.076, and 0.082 p.u., as shown in Table 4. Additionally, the suggested restoration strategy respects the voltage constraint in the 33-bus network since these values are smaller than the permissible voltage variance. Additionally, the MAC has a maximum value of zero. It means that distribution lines become overloaded for MAC (0) and when the line current limit is exceeded. Throughout the restoration procedure, the MAC values for fault locations 18, 19, 27, and 14 are 0.021, 0.036, 0.011, and 0.028 p.u., respectively. As a result, these figures are positive, and the suggested restoration mechanism meets the line current limit of the 33-bus network.

B) *CNMG overloading management*: To examine CNMG overloading management, two EV charging schemes based on [[28], [29], [30], [31], [32],[60], [61], [66], [67], [68]] are presented:

**Strategy I**: EVs begin charging operation once connected to the network, and are disconnected from the network after the battery is fully charged.

**Strategy 2**: EVs are charged depending on energy prices to achieve low charging costs and network operating constraints, allowing EVs to penetrate the network at a higher pace.

**Fig. 5:**
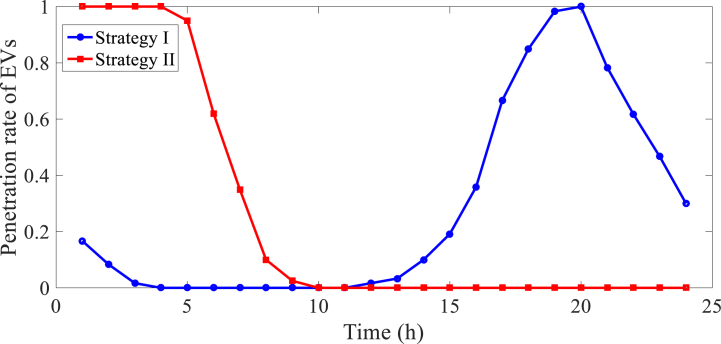
Profile of EVs penetration rate for different charging models.

EVs were charged using methods I and II at peak and low demand hours, respectively, because of [32]. Consequently, the daily EV penetration rate curve for the recommended solutions based on the results of [32] is shown in Fig. 5. Also shown in Ref. [69] are the load, solar, and wind turbine profiles. The findings from this section are listed in Table 5. Table 5 provides the overload power and lower stored energy (LSE) of MGs for procedures I and II. These values were calculated using Eqs. (14)–(16). (16). Between 20:00 and 21:00, strategy I's power is exhausted for MG, which is situated in bus 25. According to Fig. 5, the network's peak demand hours are from 17:00 to 22:00 [69] since EVs are charged during these times. Additionally, bus 25 [58] has a significant MG load between 20:00 and 21:00 due to the large number of EVs and MG. Table 5 however indicates that the reduced stored energy in MGs at buses 23, 28, and 30 is more than 40 kWh. Therefore, it is anticipated that power that has been stored in other MGs would provide the surplus power in MG 25. Additionally, bus 25 fills up with passengers for MG at around 3:00 p.m. as part of plan II. Because, according to Fig. 5, the bulk of EVs were charged between 1:00 and 7:00 a.m. Despite the fact that a single MG has only around 8 kW of overloading power, the combined LSE of all the MGs in buses 23, 28, and 30 is more over 200 kWh. As a result, the overloaded power of the MG in bus 25 may be supplied by the stored power of these MGs. Table 5 displays the outcomes of the CNMG overloading management. This table indicates that the energy not provided (ENS) or overloaded energy in MG at bus 25 is 8.09 kWh for Strategy II and 112.5 kWh for Strategy I, respectively. A side note: EV charging management may result in a lower ENS when compared to EV charging mishandling. Additionally, the overall LSE in other MGs for methods I and II is 177 and 222.9 kWh, respectively. This indicates that the phrase may be used to describe a range of strategies. For instance, the ENS of the MGs in buses 25 may be accepted by the MGs in buses 23, 28, and 30. In accordance with Table 5, MGs in buses 23 and 28 could acquire the ENS of MGs in bus 25, whereas in bus 23 MGs could obtain the ENS of MGs in bus 25 for strategy II. In order to attain the lowest energy not provided, the suggested technique is thus more suited for regulating overloading in CNMGs.

**Table 5:** CNMGs operation results.

EVs charging model		I			II		
Overloaded MG without considering CNMGs management	MG in bus	25			25		
	Period	20:00–21:00			3:00		
	ENS (kWh)	112.5			8.09		
Non-overloaded MG without considering CNMGs management	MG in bus	23	28	30	23	28	30
	LSE (kWh)	55.7	81.2	40.1	65.9	91.3	65.7
	Total LSE (kWh)	177			222.9		
Situation of overloaded MG with considering CNMGs management	ENS (kWh)	0			0		

C) *Network restoration using CNMGs*: For EV charging methods I and II in CNMGs, this analysis assumes that a failure happens in zone 24 or bus 24 at 20:00 and 30:00, respectively. Zone 24 and FC Trips define FZA as such. S23 and S24 open as a result, cutting off zone 24 from the 33-bus network. Zones 2, 3, and 23 in the upstream zone of FZA are thereafter reopened following FC's notice. Therefore, FZA starts the restoration process by sending an RFI message via bus 25 to DZA or zone 25. According to Table 5, Bus 25, which is occupied for strategy I and II during the hours of 20:00 and 30:00, has an MG and is full during those hours. When MG requests bus 25 in response, DZA sends an RFI message to OMZA to let them know. In order for the MGs on buses 23, 28, and 30 to give their excess power, the OMZA also sends RFI signals to NOMZA, or buses 23, 28, and 30. But because the switches on S23 and S24 are open, MG in Bus 23 won't get too much power from MG 25. The connection between buses 25 and 29 is also the available tie line as the overloaded power of the MG in bus 25 should be supplied by stored power from other MGs. FZA sends the CFP message to ZTA in bus 29 so that ZTA may determine its permissible power based on bus voltage and line current limits as well as the data, i.e., Vh, Vmin, Zh, and Iava, from HZA in buses 1 to 6, 26, 27, 28, 30, 31, 32, and 33. FZA instructs ZTA in bus 29 and MZA in buses 25 and 30 to shut their respective switches, s41, s42, s44, and s46, based on the information shown in Table 6. Furthermore, Table 6 shows that methods I and II's maximum voltage fluctuations are both less than 0.1 p.u. and that the MAC is positive. Therefore, the suggested restoration strategy could be sufficient to meet the network's operational needs. The optimal charge of EVs needs to optimization model. To run the optimal formulation in network, network needs to smart devices or alghorithm[[70], [71], [72], [73]].

**Table 6:** Results of coupling of the CNMGs management and restoration algorithm.

EVs charging model	Faulted bus	Bus of DZA	Bus of OMZA	Load not supplied (kVA) of OMZA	Load not supplied (kVA) of DZA	Available MG (NOMZA) in bus	Available tie line
I	24	25	25	69.9	69.9	28, 30	(29, 25)
II	24	25	25	8.09	8.09	28, 30	(29, 25)
EVs charging model	Optimal NOMZA	Optimal tie line	Switches in open mode	Switches in close mode	Total load not supplied without considering FZA load (kVA)	MAC (p.u.)	Maximum voltage deviation (p.u.)
I	30	(29, 25)	s23, s24	s41, s42, s44, s46	0	0.0088	0.0697
II	30	(29, 25)	s23, s24	s41, s42, s44, s46	0	0.0088	0.0697

## 3 Conclusions

The decentralized strategy based on MAS is used for distribution network restoration with considering CNMGs. In the CNMG, the overloading management uses with EV, RES, BSS and load model for each MG. In addition, four zone agents are used for the restoration process and one zone agent for overloading management of CNMGs, where all zone agents are coordinated together. It should be noted that the MAS approach can be used to obtain a desirable restoration path considering different zone agents dispersed throughout the system, and it can also be used to manage MGs overloaded by an unbalanced situation between RES, BSS, EV, and load by interconnecting these MGs to neighboring non-overloaded MGs. Finally, the suggested restoration approach may satisfy bus voltage and line current constraints in the distribution system in the case of a fault, according to numerical findings. Furthermore, this technology may be used to access low energy that is not available in the distribution network or MGs. Based on the numerical findings, it was shown that the suggested restoration plan may provide the most efficient means of delivering the loads with the fewest switching operations, even in cases when the fault arises at the network's worst location. Additionally, during a failure, it can maintain a voltage magnitude of greater than 0.9 p.u. and set aside a certain amount of free capacity for the distribution lines. Compared to non-smart charging, the shutdown rate of EVs in the smart charging system is around 104 kWh, or 93% lower, if the CNMG strategy is not used. However, 0% downtime for EVs is possible when the CNMG method is used to both smart and non-smart charging schemes. The proposed plan does not account for load, renewable resource, or electric vehicle uncertainties. This might make the resulting solution less reliable. In order to address this problem, the suggested strategy that takes uncertainty modeling into account is seen as future work. Furthermore, demand-side management reduces energy consumption and has a favorable influence on network technical and economic indicators. As a result, the proposed plan is seen as a future trend when considering demand side management.

## Institutional review board statement

Not applicable.

## Informed consent statement

Not applicable.

## Data availability statement

All data generated or analyzed during this study are included in this published article.

## CRediT authorship contribution statement

**Qi Cui:** Investigation. **Feng Liu:** Investigation.

## Declaration of competing interest

The authors declare that they have no known competing financial interests or personal relationships that could have appeared to influence the work reported in this paper.
